# Supplementary material for: Tuning Thermal, Morphological, and Physicochemical Properties of Thermoplastic Polyurethanes (TPUs) by the 1,4-Butanediol (BDO)/Dipropylene Glycol (DPG) Ratio
Source: Polymers (Basel). 2022 Aug 3;14(15):3164. doi: 10.3390/polym14153164 (PMC9371192; doi:10.3390/polym14153164)
Supplement: Supplementary file 1 [file polymers-14-03164-s001.zip › polymers-1825796-supplementary.pdf]

Supplementary Materials

This supplementary file shows the results of the mathematical deconvolutions of the FTIR spectra. Figure S1 are shown (a) dry polyester polyol with 2000g.mol^{-1}, (b) dry polyester polyol with 1000g.mol^{-1}, (c) dry polyester polyol blend, (d) polyester polyol blend after immersion in water, and (d) polyester polyol blend after immersion in ammonium solution. Table S1 shows the FTIR assignments and bands percentage areas of the carbonyl-stretching region of the soft segment (PM). FTIR of TPU samples are show in Fig S2 (a). The example of FTIR deconvolution (R^2>0 .999) of spectra of the carbonyl-stretching region of TPU (b) DBO and (c) DPG are shown in Figure S2. The results of the area for calculations in Table 2 are shown in Table S2.

DSC thermographs of the pure polyol blend and reactions products MDI-BDO, MDI-DPG, and MDI-BDO/DPG 50/50 are shown in Figure S3. These data were used to identify the thermal transitions of the respective HS and SS phases. Theoretical and experimental invariant data of the TPUs are shown in Figure S4. Figure S5 shows the hard and soft domain distribution obtained by GRF calculation. Finally, Figure S6 shows the tensile at 100% of deformation before hydrolysis and property retention.

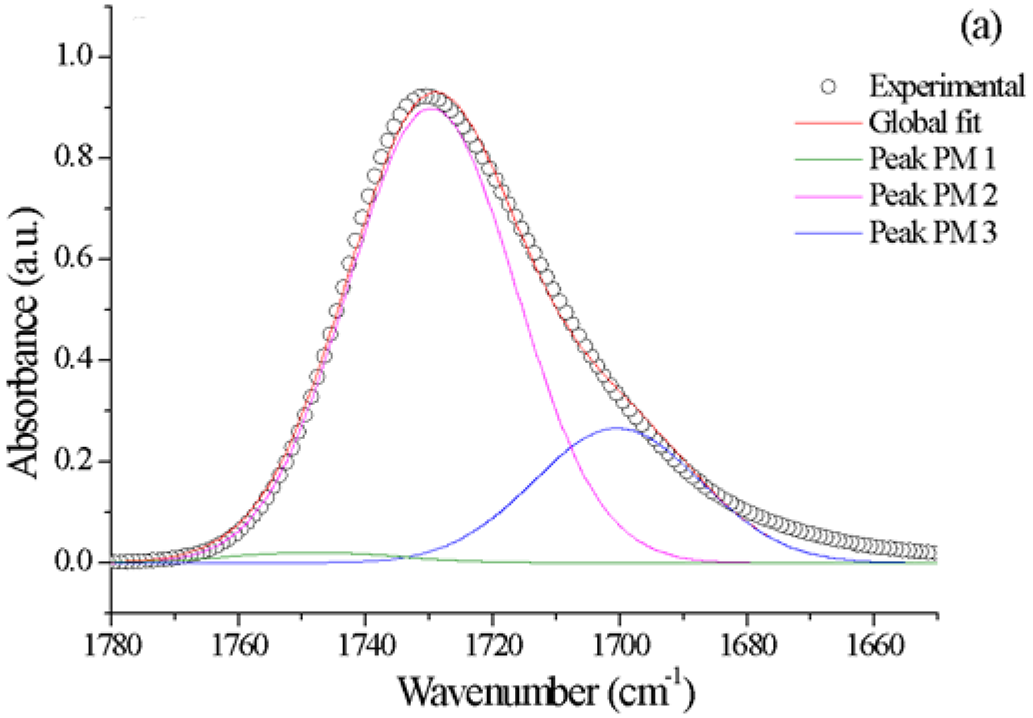

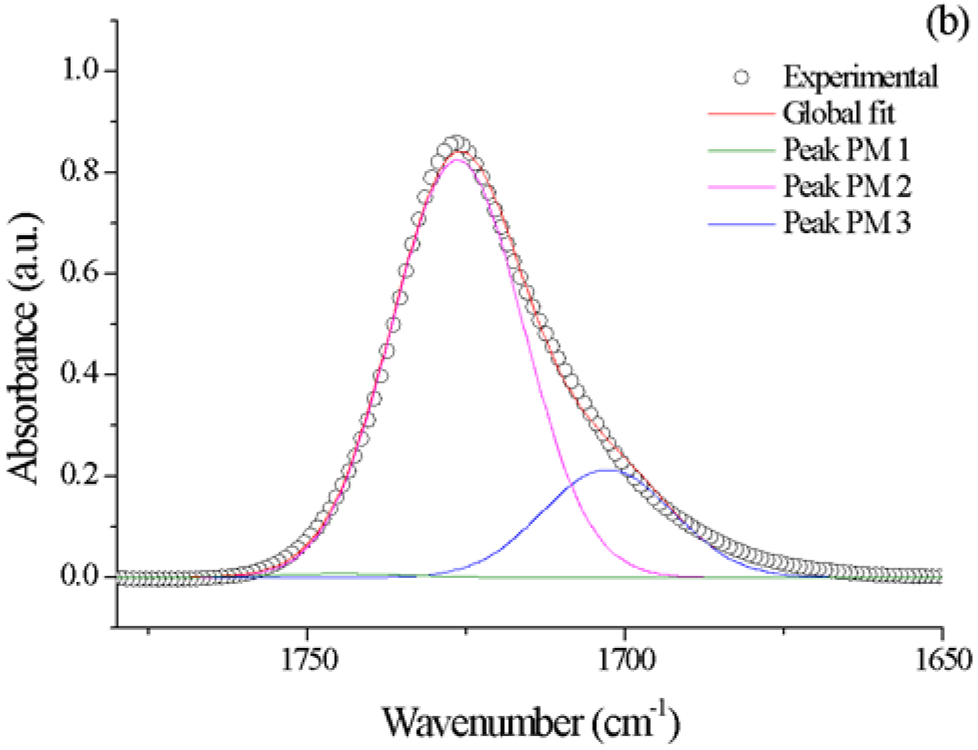
(b)
Experimental
Global fit
Peak PM 1
Peak PM 2
Peak PM 3
Absorbance (a.u.)
1.0
0.8
0.6
0.4
0.2
0.0
1750
1700
1650
Wavenumber (cm-1)

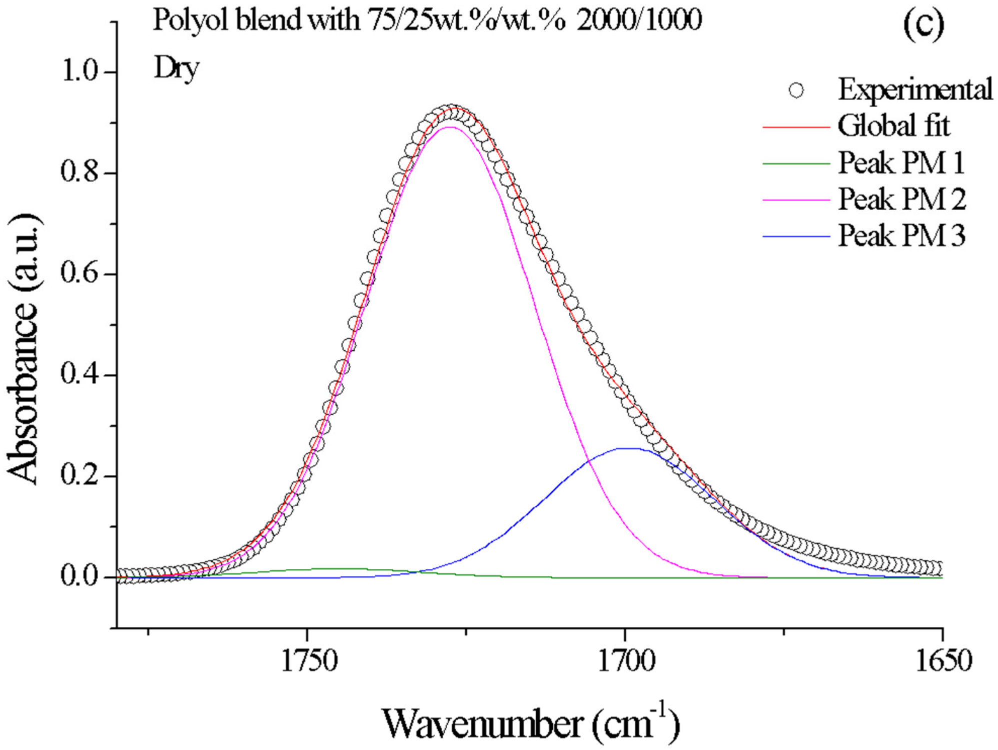
Polyol blend with 75/25wt.%/wt.% 2000/1000
Dry
(c)
Experimental
Global fit
Peak PM 1
Peak PM 2
Peak PM 3
Absorbance (a.u.)
1.0
0.8
0.6
0.4
0.2
0.0
1750
1700
1650
Wavenumber (cm-1)

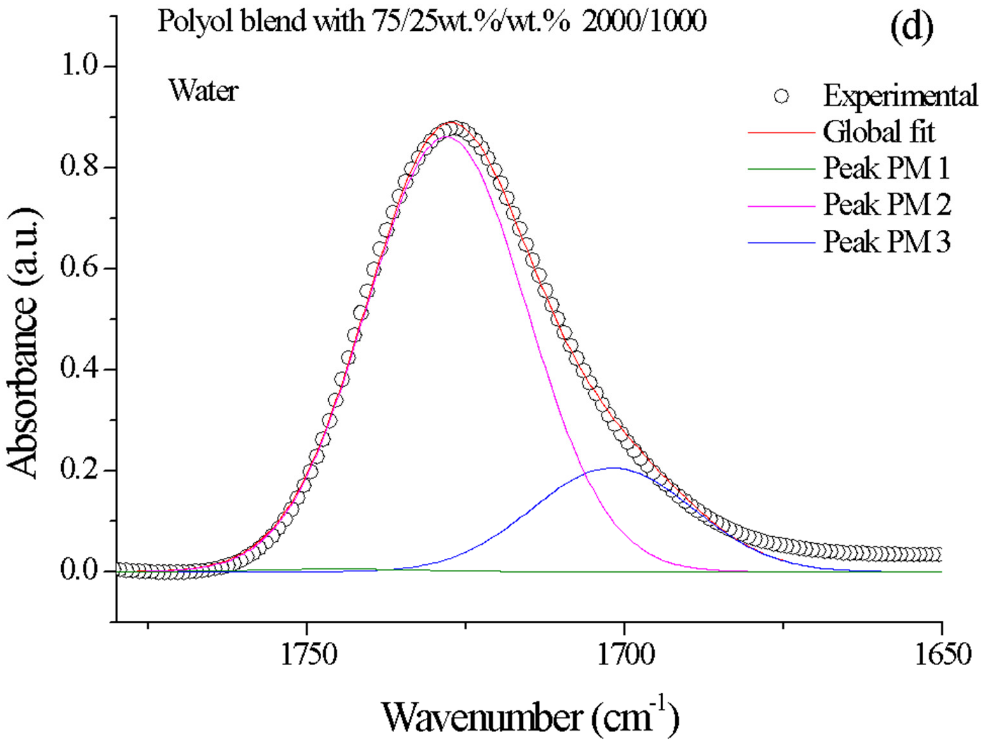

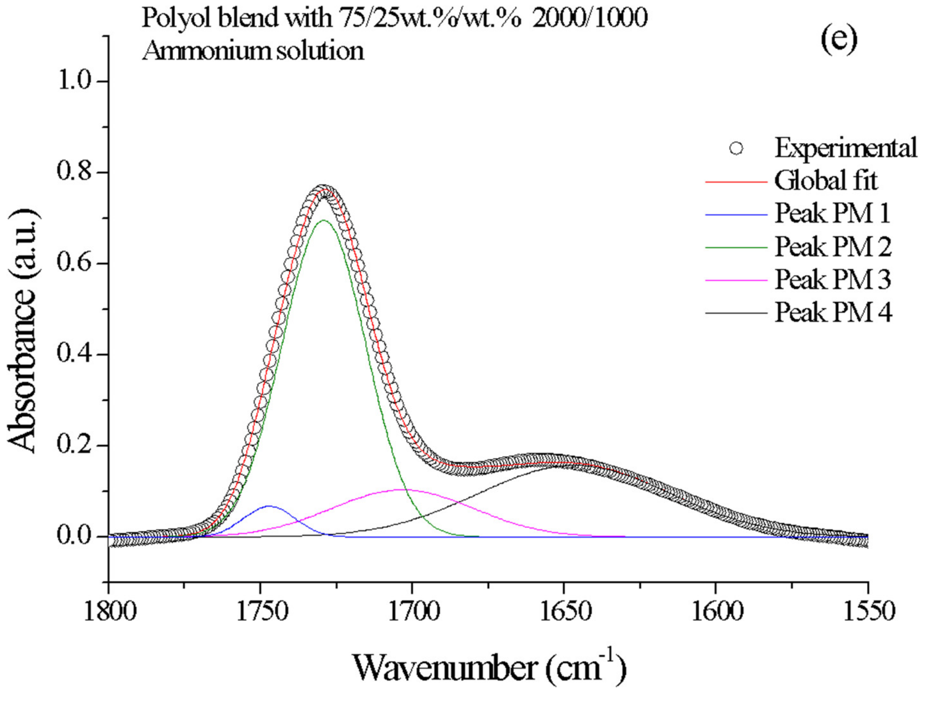

Figure S1. Deconvolution of FTIR spectra of the carbonyl stretching region of soft segment (PM): (a) dry polyester polyol with 2000g.mol^{-1}, (b) dry polyester polyol with 1000g.mol^{-1}, (c) dry polyester polyol blend, (d) polyester polyol blend after immersion in water and (d) polyester polyol blend after immersion in ammonium solution.

Table S1. FTIR assignments and bands percentage areas of carbonyl stretching region of the soft segment (PM).

Sample	Band 1 (PM)		Band 2 (PM)		Band 3 (PM)		Band 4 (PM)	
	Peak center (cm^{-1})	Area (%)	Peak center (cm^{-1})	Area (%)	Peak Center (cm^{-1})	Area (%)	Peak Center (cm^{-1})	Area (%)

Polyol with 2000 g.mol-1	1745	1.8	1729	75.8	1700	22.4	-	-
Polyol with 1000 g.mol-1	1745	0.7	1726	78.9	1702	20.4	-	-
Polyol blend 75/25	1745	1.5	1727	76.5	1700	22.0	-	-
Polyol blend 75/25 in water	1745	0.5	1728	80.3	1701	19.2	-	-
Polyol blend 75/25 in ammonium solution	1747	1.4	1729	55.4	1701	12.7	1650	30.5

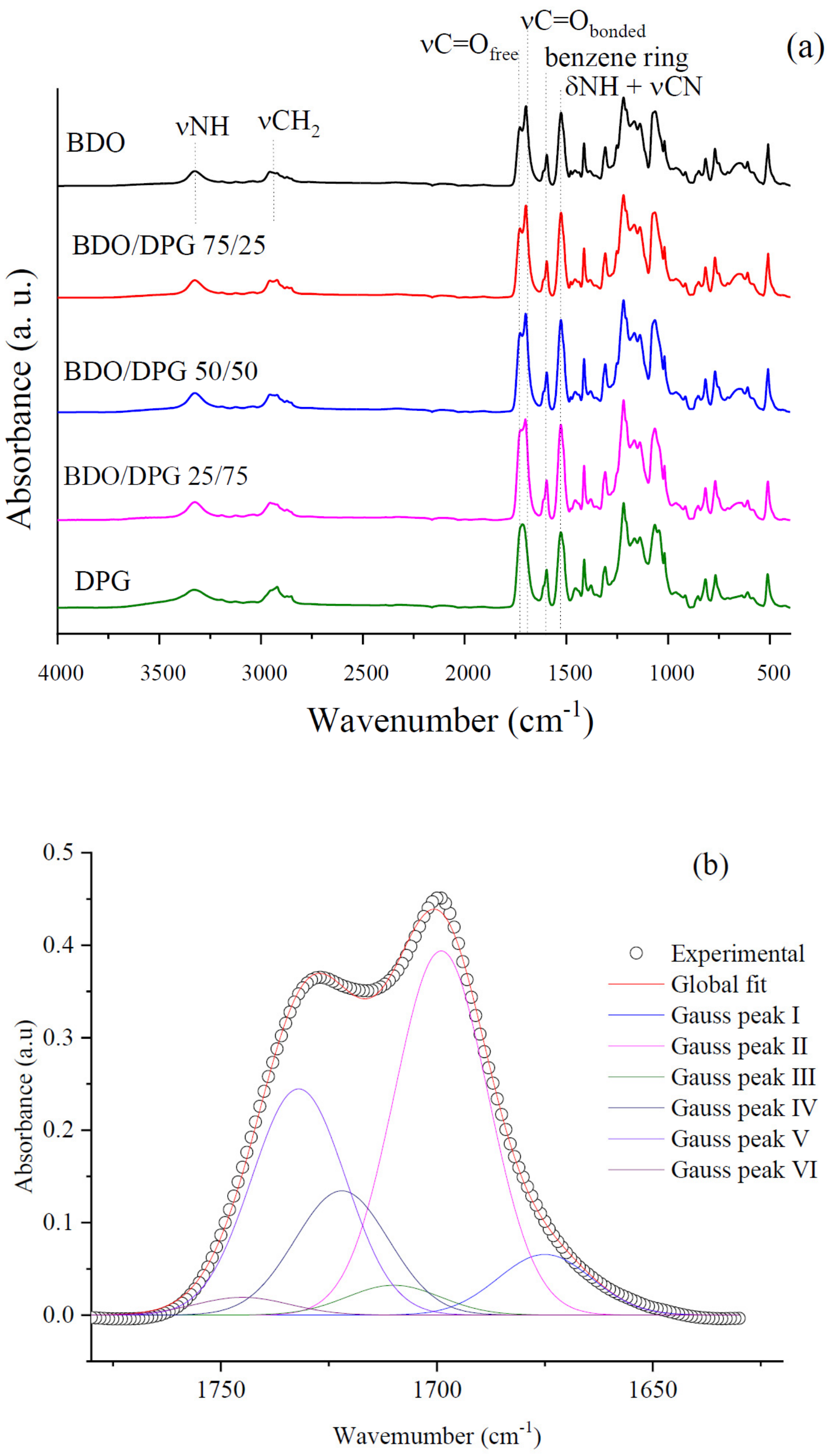

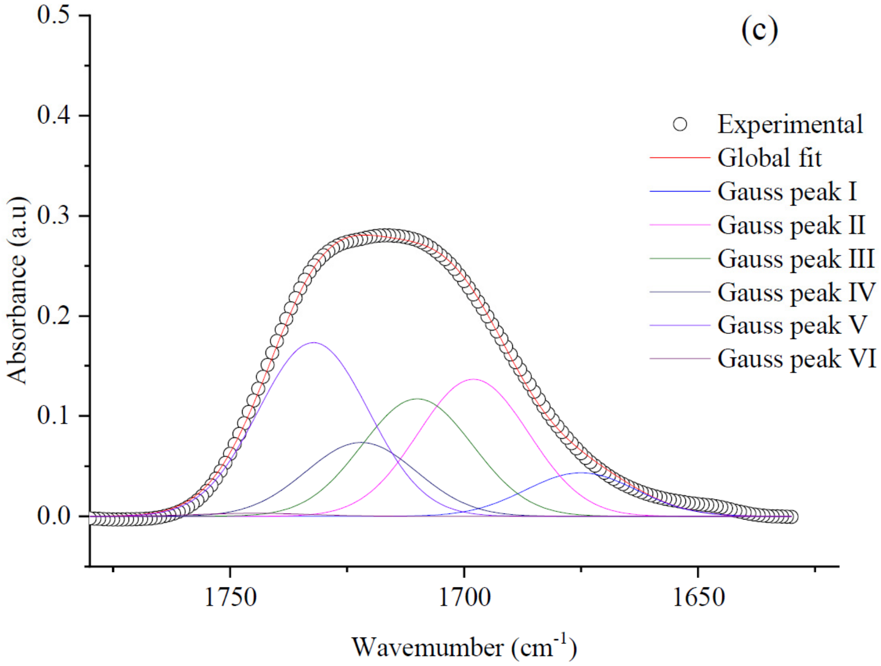

Figure S2. FTIR spectra pf the TPUs (a), example of FTIR deconvolution ($R^2 > 0.999$) of spectra of the carbonyl-stretching region of TPU (b) DBO, and (c) DPG.

Table S2. FTIR assignments and bands percentage areas of TPUs.

FTIR Band	BDO	BDO/DGP 75/25	BDO/DGP 50/50	BDO/DGP 25/75	DPG
	% area				
I	10.7	8.4	7.0	5.6	7.1
II	44.5	42.0	43.2	38.6	25.8
III	2.7	7.7	5.3	10.5	21.1
IV	13.9	12.3	15.1	14.0	14.1
V	24.8	26.0	26.8	29.8	39.1
IV	3.4	3.5	2.6	1.4	2.4

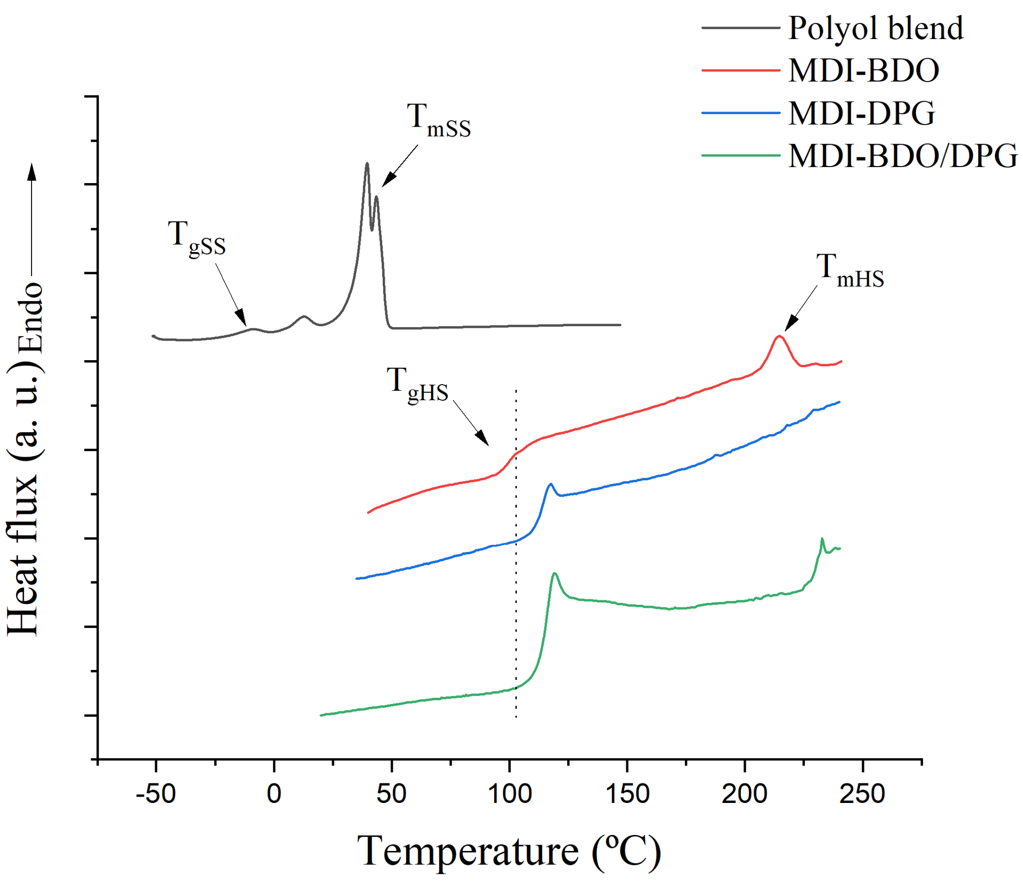

Figure S3. DSC data for pure polyol blend and reactions products MDI-BDO, MDI-DPG, and MDI-BDO/DPG 50/50.

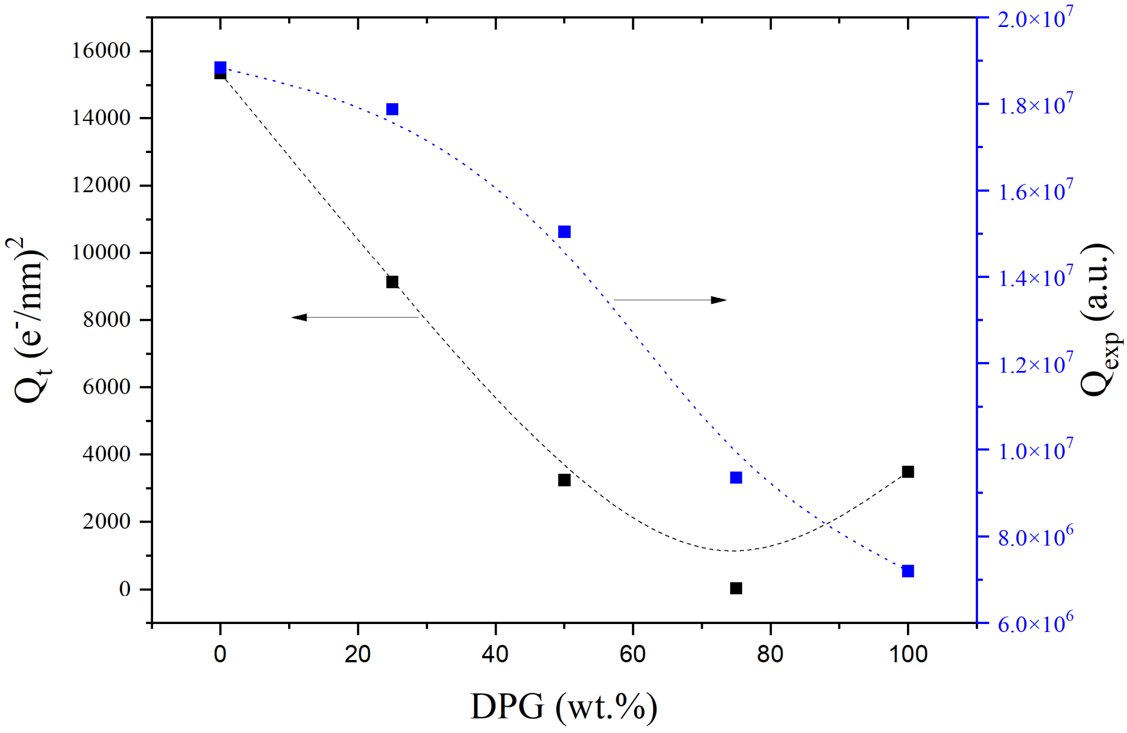

Figure S4. Experimental e theoretical invariant data.

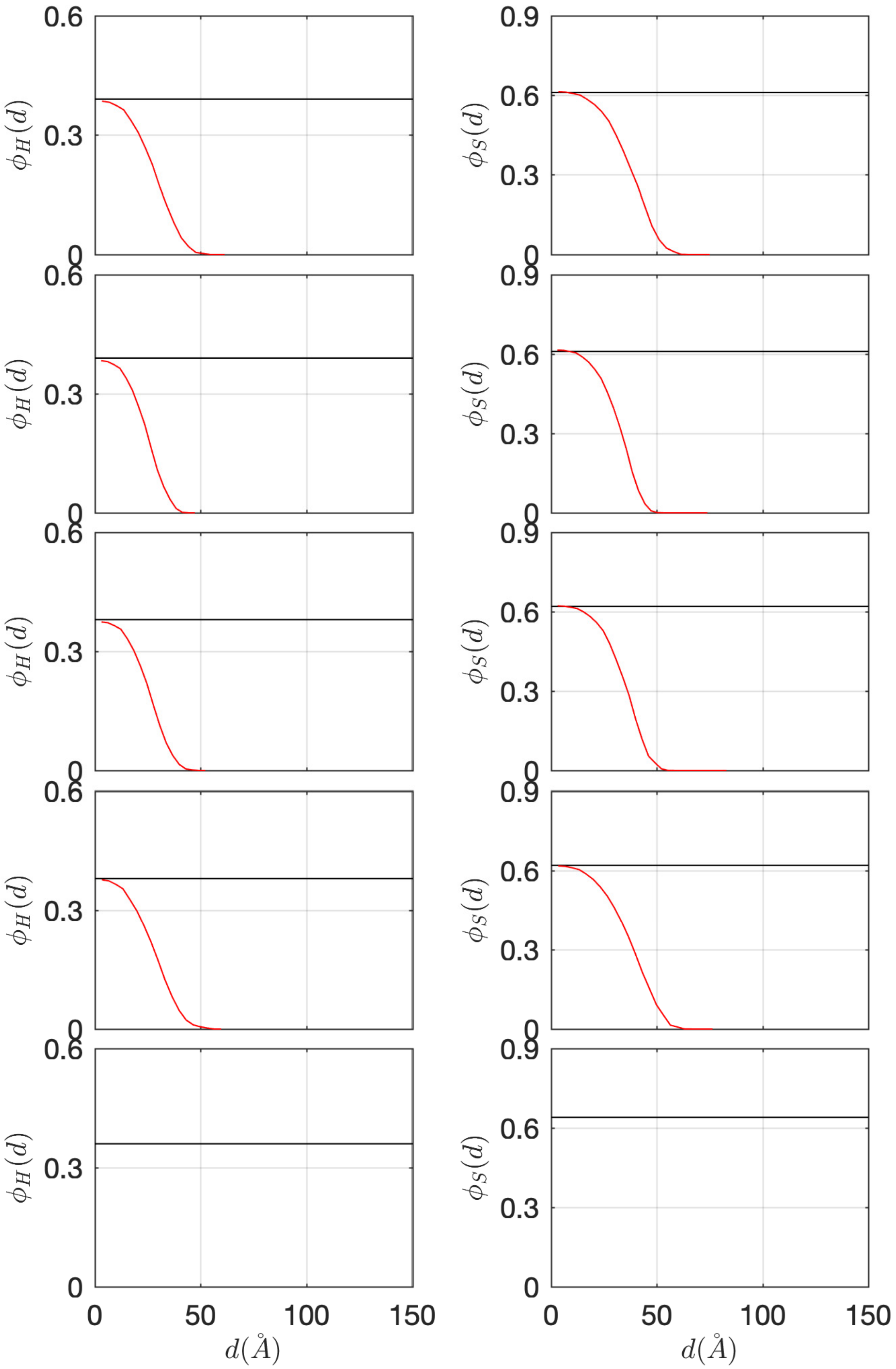

Figure S5. Hard and soft domains distributions obtained by GRF model.

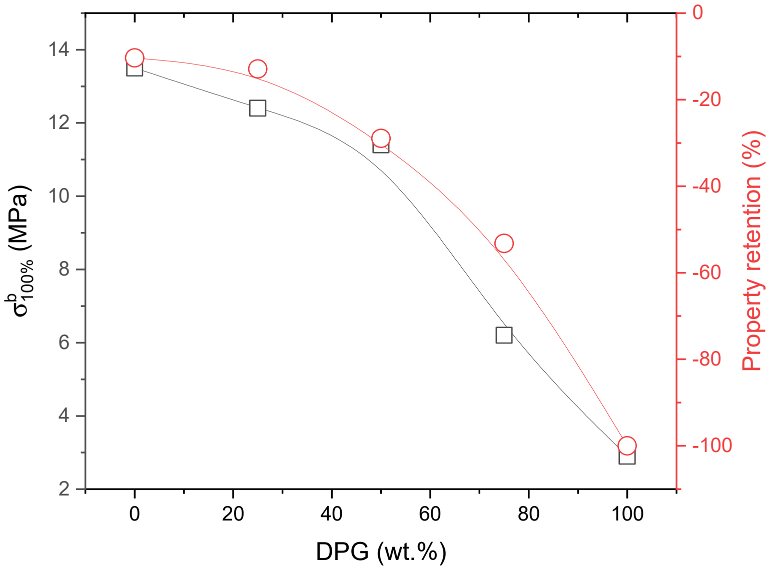

Figure S6. Tensile at 100% of deformation, σ^{b}100% before hydrolysis and property retention as a function of DPG content.
